# Sustained NF-κB Activation and Inhibition in β-Cells Have Minimal Effects on Function and Islet Transplant Outcomes

**DOI:** 10.1371/journal.pone.0077452

**Published:** 2013-10-18

**Authors:** Aileen J. F. King, Yongjing Guo, Dongsheng Cai, Jennifer Hollister-Lock, Brooke Morris, Alison Salvatori, John A. Corbett, Susan Bonner-Weir, Steven E. Shoelson, Gordon C. Weir

**Affiliations:** 1 Diabetes Research Group, King's College London, London, United Kingdom; 2 Section on Islet Cell and Regenerative Biology, Joslin Diabetes Center, Harvard Medical School, Boston, Massachusetts, United States of America; 3 Section on Pathophysiology and Molecular Pharmacology, Joslin Diabetes Center, Harvard Medical School, Boston, Massachusetts, United States of America; 4 Department of Pharmacology & Physiology, Saint Louis University, St. Louis, Missouri, United States of America; 5 Department of Biochemistry, Medical College of Wisconsin, Milwaukee, Wisconsin, United States of America; Children's Hospital Boston/Harvard Medical School, United States of America

## Abstract

The activation of the transcription factor NF-κB leads to changes in expression of many genes in pancreatic β-cells. However, the role of NF-κB activation in islet transplantation has not been fully elucidated. The aim of the present study was to investigate whether the state of NF-κB activation would influence the outcome of islet transplantation. Transgenic mice expressing a dominant active IKKβ (constitutively active) or a non-degradable form of IκBα (constitutive inhibition) under control of the rat insulin promoter were generated. Islets from these mice were transplanted into streptozotocin diabetic mice in suboptimal numbers. Further, the effects of salicylate (an inhibitor of NF-κB) treatment of normal islets prior to transplantation, and the effects of salicylate administration to mice prior to and after islet implantation were evaluated. Transplantation outcomes were not affected using islets expressing a non-degradable form of IκBα when compared to wild type controls. However, the transplantation outcomes using islets isolated from mice expressing a constitutively active mutant of NF-κB were marginally worse, although no aberrations of islet function *in vitro* could be detected. Salicylate treatment of normal islets or mice had no effect on transplantation outcome. The current study draws attention to the complexities of NF-κB in pancreatic beta cells by suggesting that they can adapt with normal or near normal function to both chronic activation and inhibition of this important transcription factor.

## Introduction

Inflammatory cytokines such as INF-γ, TNF-α and IL-1 have been implicated in the autoimmune destruction of pancreatic β-cells in type 1 diabetes [1]. Since NF-κB is both activated by these cytokines, and drives their expression, considerable interest has been focused on NF-κB in β-cells [2], [3]. But the situation is complex because NF-κB may increase the expression of both proapoptotic and antiapoptotic genes, and patterns of gene expression may vary depending on context and cell type. In β-cells, cytokine-induced activation of NF-κB has been associated with increased expression of inflammatory proteins such as iNOS and COX-2, and nitric oxide (the product of iNOS) has been implicated in IL-1β-induced β-cell death [4], [5]. NF-κB activation has also been associated with the enhanced expression of proapoptotic and protective genes [6]–[8]. *In vitro* studies have shown that the inhibition of NF-κB can protect beta cells against cytokine-induced death [9]–[11]. However, others have suggested that NF-κB activation could play a protective role preventing TNF-induced β-cell apoptosis [12]. Indeed, it has been suggested that NF-κB may play a biphasic role in cytokine-induced β-cell death, by initially protecting the β-cells before leading to apoptosis [13]. It has also been recently suggested that NF-κB may act as an antiapoptotic factor in normoxic conditions but act as an apoptotic factor in hypoxic conditions [14]. Studies have shown that genetically modified mice with disrupted NF-κB may be resistant to β-cell toxins, such as multiple low-dose streptozotocin injections [15], [16]. In transplantation settings it has been suggested that acute inhibition of NF-κB can improve islet transplantation outcome [14], [17]–[21].

Transplantation of islets is an important breakthrough in the treatment of Type 1 diabetes [22]. It can reverse hyperglycaemia in humans [23], but long-term success is limited [24], indicating a failure to maintain islet mass. Because NF-κB is a potentially useful therapeutic target and seems to be involved in β-cell destruction in models of diabetes, we sought to determine if the state of NF-κB activation would influence the outcome of islet transplantation.

The *in vivo* activity of NF-κB is tightly regulated by an inhibitory protein, IκBα [25] and an activating kinase, IKKβ [26]. Once proinflammatory stimuli have activated IKKβ, it phosphorylates IκBα, which is targeted for ubiquitination and proteasomal degradation. The liberated NF-κB translocates to the cell nucleus and drives transcription. To study the regulation of β-cell function by NF-κB, transgenic mice expressing a dominant active IKKβ to activate NF-κB (βIKK) or a non-degradable form of IκBα to prevent NF-κB activation (βISR) under control of the rat insulin promoter (RIP) were generated. In addition to these genetic approaches, NF-κB activity was modulated both *in vivo* and *in vitro* using salicylates [27]. Salicylate inhibits NF-κB, and forms of salicylate including salsalate are being investigated as a potential new therapeutic modality in patients with diabetes [27]–[30].

## Methods

### Ethics

The Joslin Animal Care Committee approved all animal experiments.

### Establishing transgenic mice

**Figure 1:**
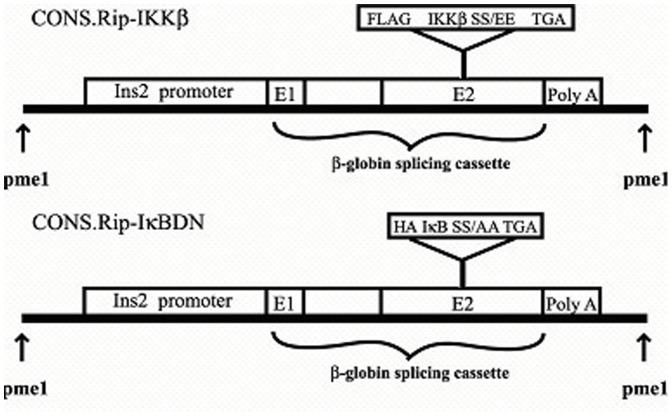
Constructs used to create βIKK and βISR mice. Dominant active IKKβ (S177/181E) or a non-degradable form of IkBα (S32/36A; super repressor) were expressed selectively in beta cells using the rat insulin 2 promoter to produce the βIKK and βISR mice, respectively. N-terminal FLAG or His tag sequences were included in exon 2 of a β-globin splicing cassette

**Figure 2:**
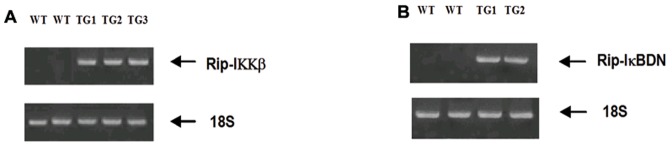
Genotyping of founder βIKK (A) and βISR (B) mice.

βIKK and βISR mice were created as described previously for both skeletal muscle (MIKK, MISR) and liver (LISR, LIKK) specific expression of dominant active IKKβ (S177/181E) and a non-degradable form of IκBα (S32/36A; super repressor), respectively [31], [32]. To produce βIKK and βISR mice, IKKβ (S177/181E) and IκBα (S32/36A), respectively, were expressed selectively in β-cells using the rat insulin 2 promoter. N-terminal FLAG or His tag sequences were included in exon 2 of a β-globin splicing cassette (Figure 1). The DNA fragments were released by Pme1 enzyme digestion and microinjected into the pronuclei of C57BL/6 oocytes, which were then implanted into pseudopregnant female mice in the Joslin Transgenic Mouse Facility. Three founders for βIKK and two for βISR were identified by tail DNA genotyping (Figure 2). These mice were created using C57BL/6 mice, and are thus 100% C57BL/6 background.

### Visualization of NF-κB in dispersed cells

NF-κB activation was assessed by immunocytochemistry. Cells in isolated islets from mice 8–12 weeks of age were dispersed using 1 mg/ml trypsin in Ca^2+^ and Mg^2+^ free Hanks' solution at 37°C for 3 min as described previously [33] washed in PBS and centrifuged onto glass slides. The cellular localization of NF-κB was examined by immunofluoresence as described previously [34] using rabbit anti nuclear p50 (1∶100 dilution, Santa Cruz, Santa Cruz, CA) and guinea-pig anti-human insulin (1∶100 dilution, DakoCytomation, Carpinteria, CA). Secondary antibodies AlexaFluor 488 goat anti guinea-pig IgG (Molecular Probes, Eugene, OR) and CY3 conjugated donkey anti-rabbit (Jackson Immunoresearch Laboratories, West Grove, PA) were used as a 1∶200 dilution and nuclei were detected using DAPI (Sigma) Slides were visualized using a Nikon Eclipse 90I.

### Visualization of NF-κB in formalin fixed pancreases and grafts

Pancreases or grafts were fixed in 4% paraformaldehyde and embedded in paraffin. Sections were stained for NF-κB using a p65 antibody (Abcam, Cambridge, MA). Briefly, slides were hydrated and triton x was applied to permeabilise the membranes. Antigen retrieval was carried out using citric acid and a pressure cooker. Slides were blocked by avidin and biotin prior to H_2_O_2_ quenching. Donkey serum (1∶50) was added for 30 min prior to the antibody, which was added overnight at 4°C. Tyramide amplification was carried out prior to the addition of the secondary antibody (Donkey anti-rabbit Alexoflour, 1∶400) for 1 hour.

### Glucose tolerance tests (GTTs) with βIKK and βISR mice

GTTs were performed on 8–12 wk old βIKK and βISR mice, as well as age and weight matched wild-type siblings. After an overnight fast, mice were injected i.p. with 2 g/kg glucose. Blood glucose levels were measured prior to the injection and at 15, 30, 60, 90 and 120 min using a glucose meter (Accu-Check; Boehringer-Mannheim Biochemicals, Indianapolis, IN) with blood obtained from a snipped tail.

### Mice receiving islet transplants

Male C57BL/6AF1 mice (Jackson Laboratories, Bar Harbor, ME) age 6-10 weeks were used as donors and recipients of islet grafts. Transgenic βIKK or βISR mice were also used as transplant donors. Recipient animals were made diabetic with a single i.p. injection of streptozotocin (Sigma, St Louis, MO) 180 mg/kg body wt, freshly dissolved in citrate buffer (pH = 4.5). Only those mice with blood glucose levels greater than 20 mmol/l were used as recipients.

### Islet Isolation

Islets were isolated using collagenase digestion followed by separation with a density gradient as previously described in detail [35]. After isolation, islets were handpicked and transplanted immediately, or cultured for 72 h in RPMI 1640+10% FCS in the absence or presence of 2 mmol/l salicylate.

### Islet transplantation

Animals were anaesthetised using 0.02 ml/g BW Avertin [2.5% (vol/vol) solution of 10 g 97% 2.2.2-tribromoethanol (Sigma) in 10 ml 2-methyl-2-butanol]. The left kidney was exposed through a lumbar incision and the kidney capsule was incised. Using a Hamilton syringe (Fisher, Pittsburg, PA) and polyethylene tubing (Cole Parmer, Vernon Hills, IL), islets were placed under the kidney capsule as previously described [36]. A suboptimal number of 150 islets were used to assess the efficacy of the transplants.

### Salicylate treatment of mice receiving islet transplants

Recipient male C57BL/6AF1 mice were injected with 180 mg/kg streptozotocin eight days before transplantation. Three days before transplantation, diabetic animals having non-fasting blood glucose levels >20 mmol/l were randomly assigned to receive water with or without salicylate. Mice were housed separately and consumption of water was calculated by weighing the water bottles every 24 h. The concentration of the salicylate was calculated based on the previous 24 h consumption of water, giving each animal approximately 30 mg salicylate/day. Salicylate administration was begun 3 days before transplantation. Mice were transplanted with 150 freshly isolated islets under the left kidney capsule. Blood glucose levels and water consumption were monitored daily. The concentration of salicylate in the water was modified as blood glucose levels fell and the mice were drinking less water. Salicylate administration was continued until the end of the study (28 days), irrespective of whether or not the animals were cured.

### Salicylate administration to cultured islets

Islets were cultured in RPMI 1640 and 10% foetal calf serum in petri dishes in groups of approximately 200 islets for 72 h as described above. The media contained a salicylate concentration of 2 mmol/l, which was changed every 24 h. For transplantation, 150 islets were handpicked and transplanted as described above. Blood glucose levels and weights were monitored weekly and i.p. GTTs were carried out 10 wk after transplantation in cured mice.

### Insulin secretion and content of islets

To study insulin secretion *in vitro*, triplicate groups of ten islets were placed in glass vials containing 250 µl Krebs-Ringer bicarbonate buffer supplemented with 10 mmol/l HEPES (Sigma-Aldrich), hereafter referred to as KRBH buffer. In addition, the KRBH was supplemented with 2 mg/ml bovine serum albumin (fraction V; MP Biomedicals Inc, Aurora, OH, USA) and 1.7 mmol/l glucose or 16.7 mmol/l glucose, for the first and second hour of incubation, respectively. Islets were either cultured in the presence or absence of 2 mmol/l salicylate for 72 h and then islets from each group were then split into two groups with or without 2 mmol/l salicylate. After the secretion experiments, the islets were pooled into groups of 30 and insulin was extracted overnight at 4°C in acid ethanol to determine insulin content. Insulin concentrations were determined by insulin ELISA (Mercodia Rat Insulin ELISA; Mercodia AB, Uppsala, Sweden).

### Glucose oxidation measurements

Islet glucose oxidation rates were determined with a previously described method [37]. Triplicate groups of ten islets were incubated in glass vials containing 100 µl Krebs-Ringer bicarbonate buffer supplemented with 10 mmol/l HEPES (Sigma-Aldrich), hereafter referred to as KRBH buffer. The KRBH was supplemented with D-[U-^14^C] glucose (0.3 mCi/mmol/l, Amersham, UK) and non-radioactive D-glucose to give final glucose concentrations of 1.7 and 16.7 mmol/l. The ^14^CO_2_ formed by cell metabolism was entrapped in hyamine and measured by liquid scintillation counting.

### Viability of islets

The viability of islets was measured using propidium iodide and Hoechst staining. Five isolated islets were analysed from each mouse with an average of 361±20 cells counted in each islet and an average of 1809±152 cells counted per animal. Islets from five βIKK and five wild-type mice were analysed in a blind fashion. Mice between 16 and 24 wk of age were matched with siblings. A mixture of 2 mg/ml propidium iodide (Sigma) and 0.5 mg/ml bisbenzimide (Hoechst 33258, Sigma) was added to the islets, which were incubated at 37°C for 15 min, then washed with PBS and put on a coverslip. Images were taken using a Zeiss Axiocam camera on a fluorescent microscope (Leica, Leitz DMRB) with a UV-2B filter and Openlab 3.0.4 software. The total number of cells living (stained blue) and dead cells (stained red) were counted using ImageJ (rsb.info.nih.gov/ij/).

### In vitro evaluation of βIKK islets

In the βIKK islets, insulin release measurements, glucose oxidation measurements and viability studies were performed in fresh islets. In addition, βIKK islets were cultured in RPMI 1640 media supplemented with 10% FCS. Islets were cultured either in control conditions (11.1 mmol/l glucose) or with a high glucose concentration (33 mmol/l), or in the presence of IL-1β (2.5 U/ml) for a period of 48 h. After this period, insulin release and content, and glucose oxidation were measured.

Real time PCR for insulin mRNA was carried out using a method previously described in detail [38], using the following primers:

insulin forward: 5′-ACAGCACCTTTGTGGTCC

insulin reverse: 5′-GGACTCAGTTGCAGTAGTTC

β-actin forward:5′-GCCCTGGCTCCTAGCACC

β-actin reverse: 5′-CCACCAATCCACACAGAGTACTTG

### Statistical analysis

Values are expressed as mean±SEM. When two groups were compared, unpaired two-tailed Student's t-test was used. When more than two groups were compared an analysis of variance (ANOVA) was used. Repeated measurement ANOVA (RM ANOVA) was used when the same groups were tested at different time-points. Two way ANOVA was used when more than one variable was being considered. If the ANOVA was significant, the Student-Newman-Keuls (SNK) post hoc test was performed.

For all comparisons, p values of less than 0.05 were considered statistically significant. All statistics were carried out using Sigmastat 3.1 (Systat Software, Erkrath, Germany).

## Results

### Characterization of NF-κB in βIKK and βISR beta cells

**Figure 3:**
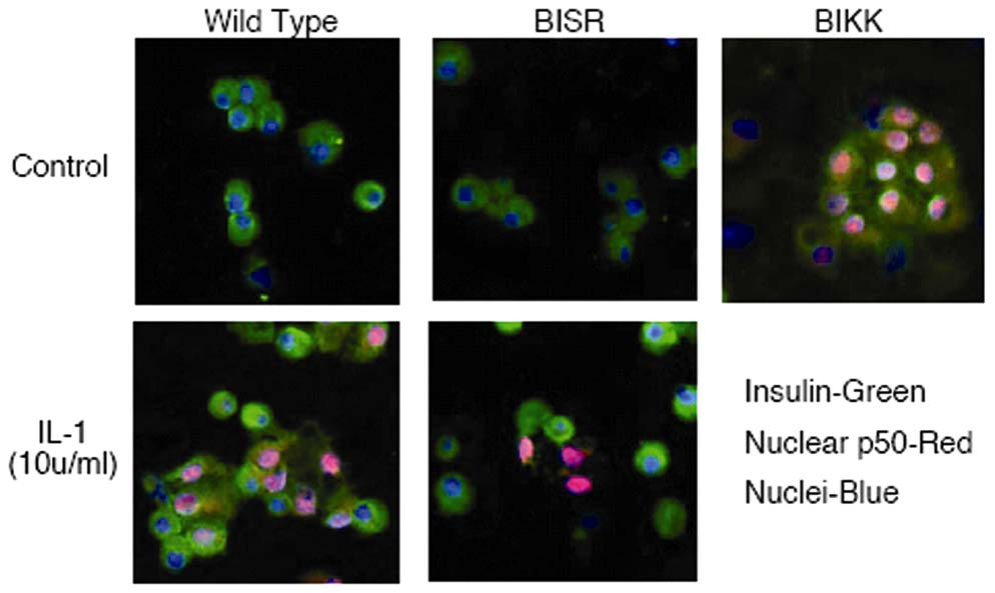
Translocation of NF-κB to the nucleus in islet cells from βIKK and βISR 8–12 week old mice in the presence or absence of 10 U/ml IL-1, using immunohistochemical detection of the NF-κB subunit p50, by an antibody specific for nuclear localized p50.

Using an antibody specific for nuclear p50, the activation state of NF-κB in β-cells of βIKK and βISR mice was evaluated by immunofluorescence. NF-κB is not present in the nucleus of insulin containing cells isolated from wild-type mice, however, following a 30 min treatment with 10 U/ml IL-1β, NF-κB is nuclear localized in ∼50% of insulin containing cells (pink, Figure 3). In islet cells isolated from βISR mice, IL-1β failed to stimulate NF-κB nuclear localization in β-cells, but did induce nuclear localization of NF-κB in some non-β-cells. NF-κB is constitutively nuclear in β-cells obtained from βIKK mice. These findings confirm the predicted activation state of NF-κB in these transgenic mice.

### Glucose tolerance of βIKK and βISR mice

**Figure 4:**
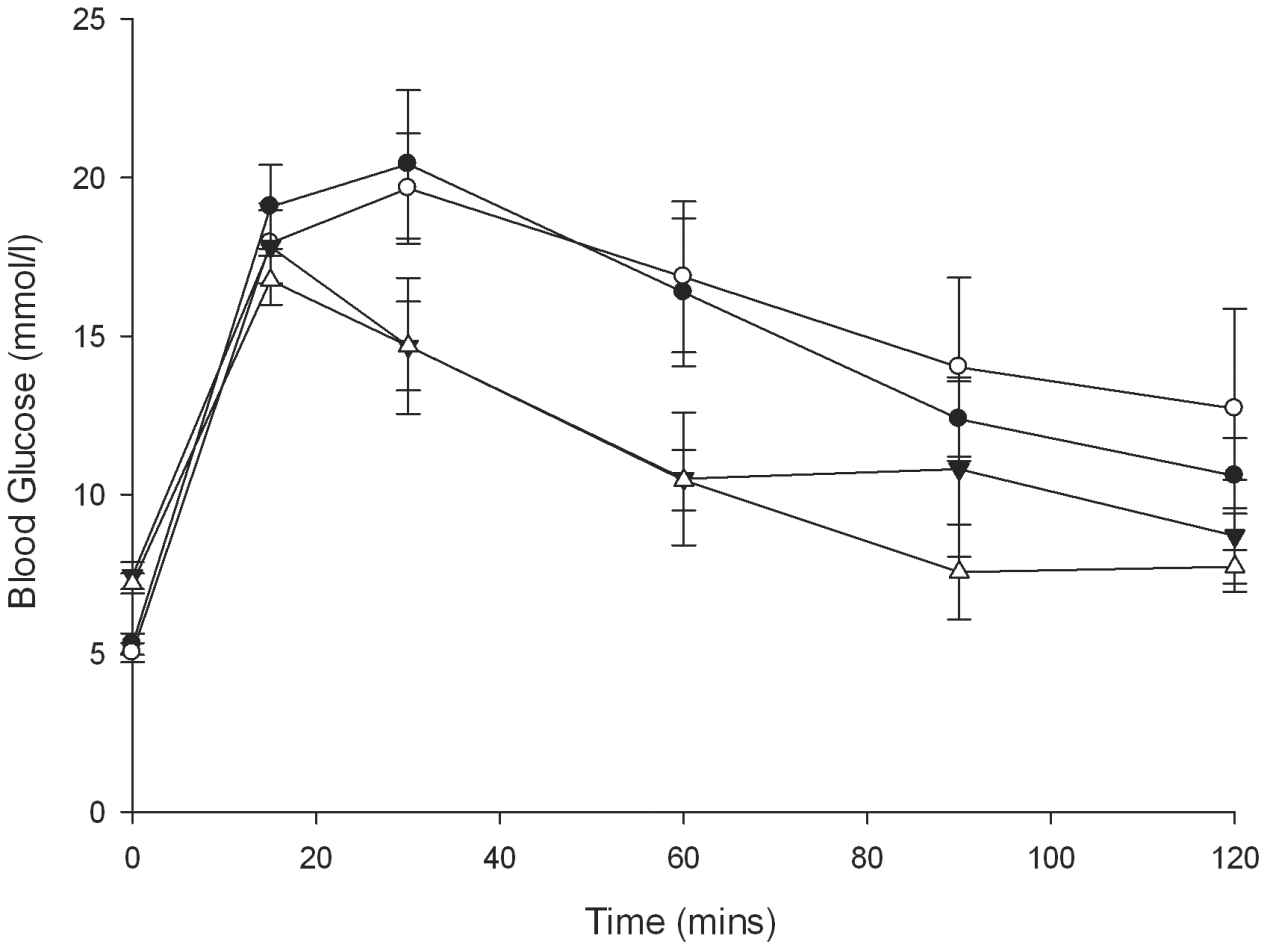
Intraperitoneal glucose tolerance tests (2 g/kg) conducted in female βISR (filled circle) vs. wt littermate control (open circle) mice (n = 6–7) and in female βIKK (filled triangle) vs. wt littermate control (open triangle) mice (n = 4–5).

There were no differences in i.p. GTTs, with both female and male βISR mice showing similar glucose clearance as wild-type weight-matched gender-matched littermates at 12–16 weeks of age (Figure 4, female mice). Lean βIKK male and female mice also show similar glucose tolerance as their wild-type weight-matched gender-matched littermates at 10–14 weeks of age (Figure 4, female mice; males not shown).

### Salicylate treatment of mice receiving islet transplants

**Figure 5:**
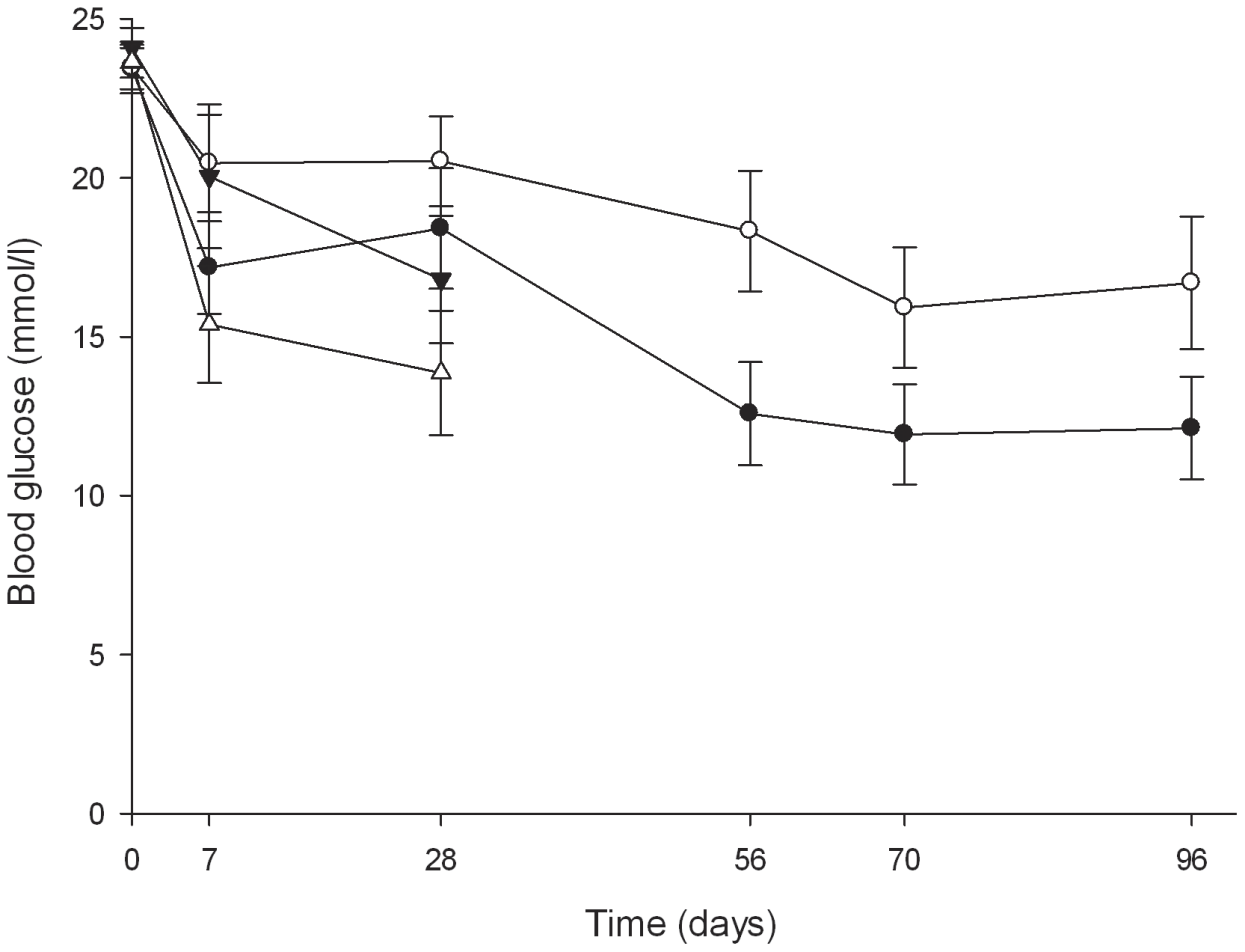
Blood glucose levels in STZ diabetic male C57BL/6AF1 mice transplanted with 150 fresh islets with administration of salicylate in the water (closed triangles) or regular water (open triangles). 2 way RM ANOVA with the factors being treatment and time; p = 0.153 for the effect of salicylate treatment on blood glucose, p<0.001 for the effect of time on blood glucose, n = 11–14. Alternatively, STZ diabetic male C57BL/6AF1 mice were transplanted with 150 islets, which had been cultured for 72 h with 2 mmol/l salicylate (closed circles) or without salicylate (open circles). 2 way RM ANOVA; p = 0.072 for the effect of salicylate treatment on blood glucose, p = 0.401 for the effect of time on blood glucose, n = 12–14.

The consumption of water during the 24 h time period prior to islet transplantation was similar in both groups of mice (18.7±1.1 ml/mouse/24 h in control mice and 18.5±2.1 in mice receiving water with salicylate). When a mouse became normoglycaemic (non-fasting blood glucose <11.1 mmol/l), its water consumption dropped to 3–5 ml per 24 h. Three of 11 salicylate-treated and 8 of 14 control mice were cured during the course of the study. The blood glucose concentrations in mice administered salicylate were not significantly different from control mice (Figure 5, 2 way RM ANOVA; p = 0.153 for salicylate treatment, p<0.001 for time passed after transplantation).

### Transplantation results from islets cultured with salicylate

**Figure 6:**
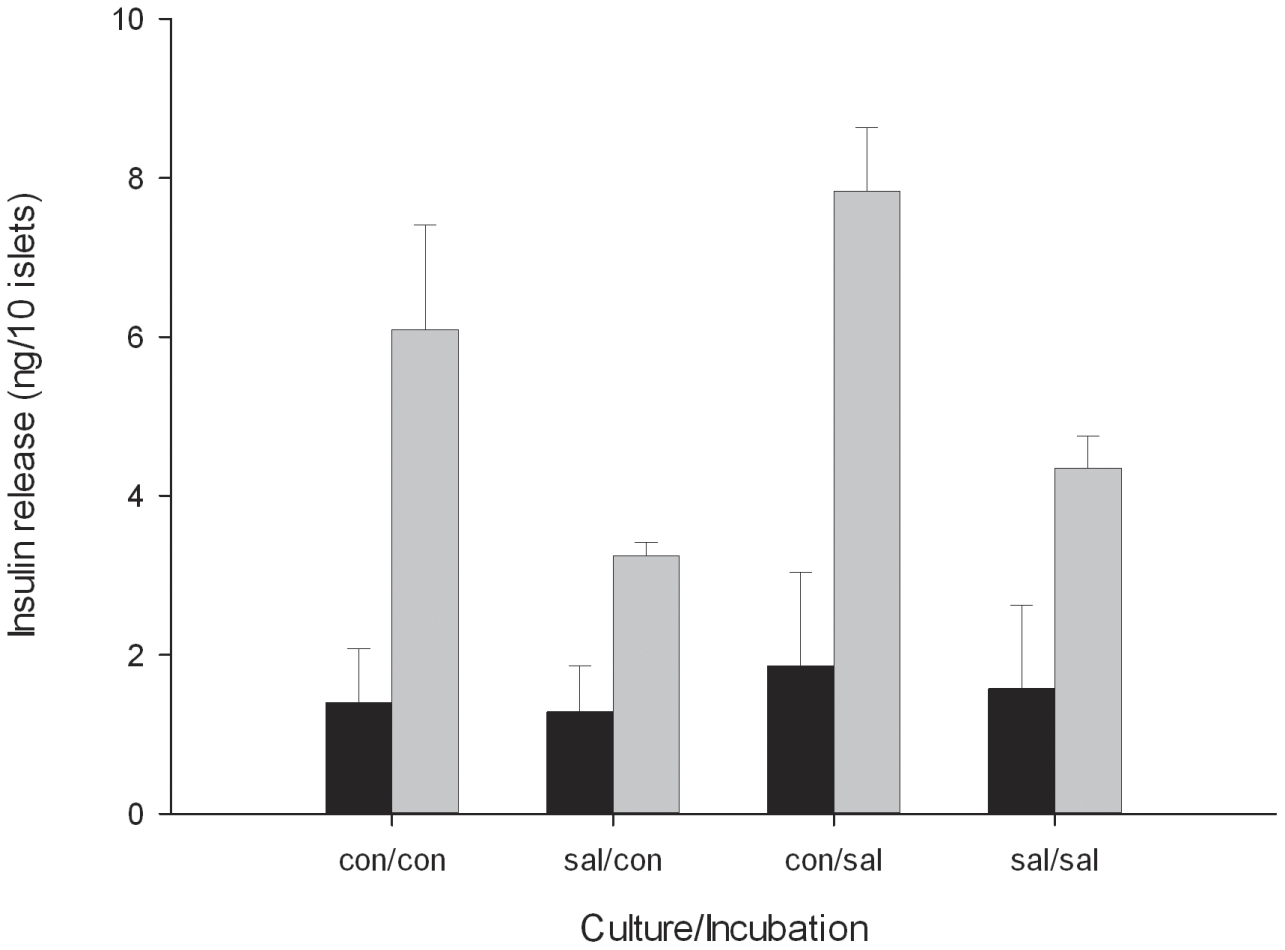
Insulin secretion in islets cultured with 2/l salicylate for 72 h and/or exposed to 2 mmol/l salicylate during the insulin release experiment (1 h at 1.7 mmol/l (black) and 1 h at 16.7 mmol/l glucose (grey)). p = 0.035, RM ANOVA with Student-Newman-Keuls (SNK) post hoc test, n =  5 separate experiments with triplicates.

When islets were cultured for 72 h with salicylate and then transplanted, the blood glucose levels of the recipient mice tended to be lower than in mice with control islets (Figure 5, two way RM ANOVA; p = 0.072 for salicylate treatment, p = 0.401 for time). Twelve weeks after transplantation, diabetes was cured in 33% (4 of 12) of control mice (non-fasting blood glucose <11.1 mmol/l). By contrast, 64% (9 of 14) mice that had received salicylate-cultured islets had been cured. After culturing the islets with salicylate for 72 h, insulin release rates at 16.7 mmol/l glucose were lower than in islets cultured identically but without salicylate (Figure 6, p = 0.035, RM ANOVA with Student-Newman-Keuls (SNK) post hoc test). Islets exposed to salicylate only during the insulin release experiment did not have different insulin release rates than the control islets (p = 0.161, RM ANOVA with Student-Newman-Keuls (SNK) post hoc test).

### Transplantation of βISR and βIKK islets

**Figure 7:**
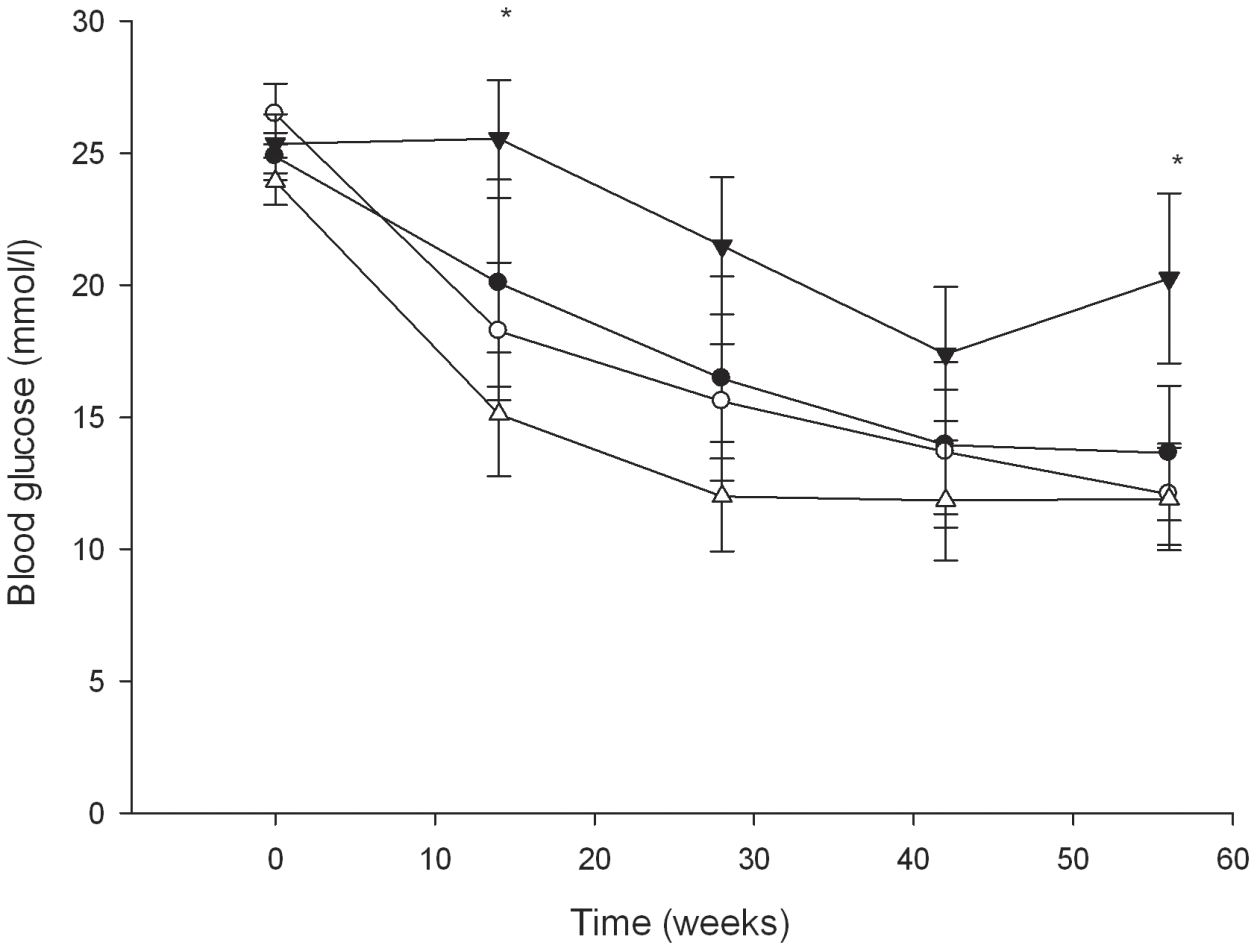
Blood glucose levels after transplantation of 150 islets from βISR mice (closed circles) or βIKK mice (closed triangles) and their respective wild-type littermates (open symbols) into STZ diabetic male C57BL/6AF1 recipients. n = 8–13. * = p<0.05, t-test vs wild-type littermates of βIKK mice, n = 12–13.

**Figure 8:**
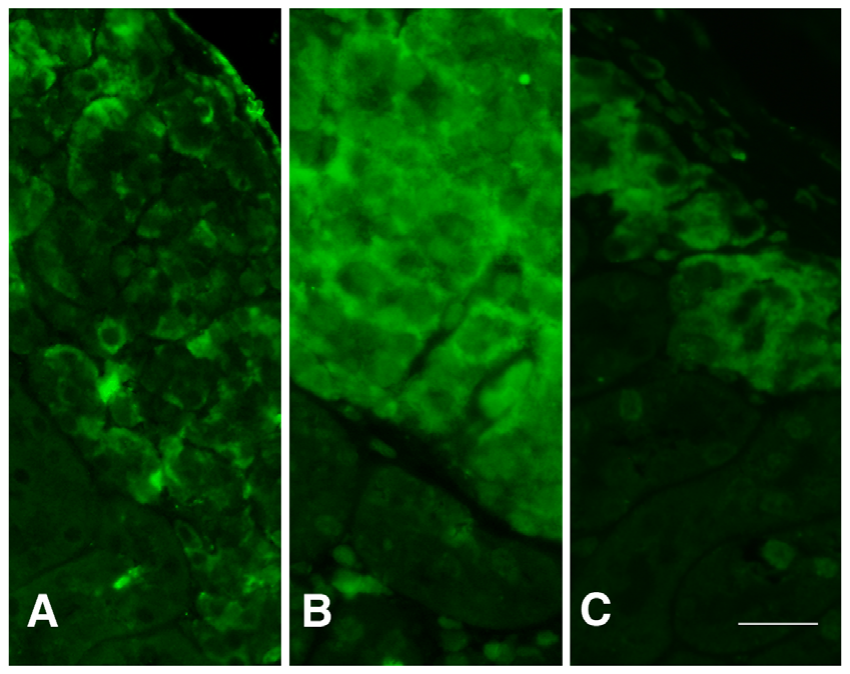
NF-κB localization in βIKK and wild-type islets 98 days after transplantation. In wild-type islets (panel A and C), the NF-κB localization is cytoplasmic whereas in βIKK islets (panel B) the localization is nuclear. Scale bar is 20 µm.

Transplantation of βISR islets resulted in similar blood glucose levels as transplantation of islets from wild-type littermates (Figure 7). After transplantation of βIKK islets, the recipient mice had higher blood glucose levels at 14 and 56 days compared with mice that received islets from wild-type littermates (Figure 7). At day 56, 64% of mice transplanted with βIKK islets remained overtly diabetic (blood glucose >20 mmol/l) as compared to 23% of mice transplanted with wild-type islets. In mice transplanted with βISR islets, 37% were overtly diabetic at day 56 compared to 27% of mice transplanted with wild-type islets. When grafts were studied for the presence of NF-κB using immunohistochemistry, it was evident that the βIKK grafts showed a higher activation of NF-κB (as demonstrated by its nuclear localization) whereas wild-type grafts showed little nuclear NF-κB staining (Figure 8).

### In vitro function of βIKK islets

To better understand why the βIKK islets were less effective in reversing hyperglycaemia, we studied their function in vitro. There were no differences in insulin release rates at 16.7 mmol/l glucose, comparing freshly isolated βIKK vs. wild-type islets (16.9±3.6 vs. 16.6±3 ng insulin/10islets/h, respectively; p = 0.72, t-test). Moreover, glucose oxidation rates were similar for freshly isolated βIKK and wild-type islets cultured either at 1.7 mmol/l glucose (25±7 and 29±4 pmol/10 islets/90 min, respectively; p = 0.74, t-test) or 16.7 mmol/l glucose (208±27 and 173±43 pmol/10islets/90 min, respectively; p = 0.51, t-test). Insulin mRNA expression was similar as well, as measured by real time PCR, in freshly isolated βIKK islets compared with wild-type islets (705±49 vs 683±72 arbitrary units when normalized to actin, respectively; t-test, p = 0.81, n = 5). However, there was a modest but significant decrease in the viability of βIKK islets directly after isolation compared with wild-type islets (89.8±0.8 vs 93.6±0.8%, p = 0.0013, t-test, n = 5).

After 48 h culture periods, GSIS (stimulation index of glucose induced insulin secretion) tended to be lower for βIKK islets than control islets, but this did not reach significance (Table 1). Culture of islets for 48 h in the presence of 2.5 U/ml IL-1β reduced GSIS to similar levels in βIKK and wild-type islets (Table 1). In addition, culture of the islets in 33 mmol/l glucose for 48 h prior to the insulin release experiment also had similar effects in both βIKK and wild-type islets.

**Table 1:** Insulin secretion from wild type (WT) and βIKK islets after culture for 48 h in 2.5 U/ml IL-1β, 33 mmol/l glucose or in control media.

Culture conditions (48 h)	Control		2.5 U/ml IL-1β		33 mmol/l glucose	
**Glucose concentration during incubation (mmol/l)**	**1.7**	**16.7**	**1.7**	**16.7**	**1.7**	**16.7**
**WT islet insulin release** (ng/10 islets/h)	2.4±0.6	16.5±5.8	5.4±2.2	9.2±2.1	12.7±1.0	28.0±5.7
**βIKK islet insulin release** (ng/10 islets/h) *(p value: t-test vs WT islets)*	3.2±1.1 (p = 0.57)	13.0±5.8 (p = 0.69)	7.3±2.5 (p = 0.75)	13.7±2.7 (p = 0.24)	11.6±3.3 (p = 0.51)	22.2±4.8 (p = 0.46)
**Stimulation index WT islets**	7.7±2.7		2.8±1.3		2.3±0.7	
**Stimulation index βIKK islets** (p *value: t-test vs WT islets)*	4.6±1.4 (p = 0.31)		3.9±1.8 (p = 0.69)		3.1±1.4 (p = 0.66)	

n = 5–7 separate experiments with triplicates.

After culturing under control conditions (RPMI 1640 with 10% FCS) for 48 h, wild-type islets contained 20.5±7.2 ng insulin/islet and BIKK islets contained 14.3±3.1 ng insulin/islet (p = 0.4, t-test, n = 5–7). After culture in 33 mmol/l glucose, there was a tendency for the βIKK islets to contain less insulin (9.1±1.9 ng/wild type islet versus 5.2±1.1 ng/βIKK islet, p = 0.09, n = 5–7). After exposure to 2.5 U/ml IL-1β, insulin content of wild-type islets was 8.1±1.6 ng/islet and in βIKK islets 10.5±2.5 ng/islet (p = 0.5, n = 5–7).

## Discussion

The surprising finding of this study is that neither chronic inhibition (βISR) nor activation (βIKK) of NF-κB in β-cells of transgenic mice led to abnormal metabolic phenotypes, indicating that in vivo β-cell function was either normal or at least compensated. This was supported by studies of islet function *in vitro* with islets from βIKK mice, in which dysfunction might have been expected. Indeed, results of measurements of GSIS, insulin content, insulin mRNA, and glucose oxidation did not differ from results in control wild-type mice. In addition, when cultured islets were exposed to IL-1β or to high glucose levels, no significant differences in insulin content between βIKK and control islets emerged.

Transplantation experiments were used to further challenge the βIKK islets. In this situation, the βIKK islets did a little less well, but it is impressive that a minimal number of 150 islets with activated NF-κB were able to cure 36% of the mice. We can conclude that chronic activation of NF-κB does not have a very damaging effect on transplanted islets. It may be that chronic activation of NF-κB in islets is different than its acute activation, or that NF-κB activities in β cells are less important than previously suggested.

Roles of NF-κB in islet transplantation appear to be complex. There is even disagreement about whether NF-κB is activated by the trauma of the isolation process; some find activation [21], [39] while others do not [40]. Given that NF-κB activation does occur, there are questions about how damaging it is because both proapoptotic and antiapoptotic factors can be generated [13], [16]. However, it has been suggested that inhibiting NF-κB prior to and immediately after islet isolation does improve islet transplantation outcome [17], [21]. Nonetheless, there are a variety of death pathways that could be independent of NF-κB such as c-jun NH2-terminal kinases (JNKs) [40] and poly(ADP-ribose) polymerase [39]. In addition, there must be adaptive changes that occur over the time period NF-κB is activated. The acute changes seen after isolation may also be different than those produced through activation by an inducible or constitutive transgene. In addition to changes induced by the isolation process, more serious trauma is inflicted during the peritransplant period such as anoxic cell death [41]. Indeed it has been recently suggested that hypoxic conditions can determine whether NF-κB is pro- or anti- apoptotic [14].

A question addressed by this study is whether inhibition of NF-κB either by genetic or by pharmacological means might protect transplanted islets. In spite of the complexities outlined above, there was reason to think that NF-κB inhibition might protect islets after isolation and/or during the peritransplant period. The βISR mice were created to provide constitutive inhibition of NF-κB. Unexpectedly, transplantation of a marginal number of βISR islets did no better than control wild type mouse islets in a syngeneic model. It is entirely possible that an acute intervention might have provided protection not seen with our chronic model, as was reported recently by Rink et al [17]. The current study does however indicate that whatever cell death occurred in this transplant situation was independent of NF-κB. However it should be noted that the situation may be different in the case of allogeneic rejection, where inhibition of NF-κB has been shown to prolong graft survival [18], [19]. Salicylate treatment provided an opportunity to test the effects of pharmacologically interfering with NF-κB. Salicylate is known to inhibit NF-κB and was previously shown to have antiapoptotic effects in human islets [11]. Treatment of recipient mice by addition of salicylate to drinking water provided no benefit, but culture of islets with salicylate prior to transplantation provided outcomes that came close to being significantly better (p  =  0.072). Insight into the issue of acute versus chronic activation may be provided by a paper in which NF-κB was conditionally blocked in a transgenic model by treatment with doxycycline for three days [16]. This was followed by impressive protection of islets from cytokine-induced apoptosis and treatment with multiple low-dose streptozotocin. In a transplantation setting, it has been recently suggested that conditional knock-out of NF-κB in islets prior to isolation and culture can improve islet transplantation outcome in intraportally implanted islets [17]. This indicates that the viability of the islets prior to transplantation is particularly important. It is interesting to note that in our study, wild-type mice showed little NF-κB staining 8 wk after implantation, which may explain why chronic inhibition of NF-κB did not seem to have any beneficial effects.

In our model in which islets were pre-cultured with salicylate, there were trends towards improved transplantation outcomes. This indicates that inhibiting NF-κB for a short period prior to islet transplantation may be beneficial. Interestingly, islets that had been cultured with salicylate showed decreased GSIS but it is likely that this effect was reversible as after implantation, 64% of the animals with salicylate-treated islets cured. A salicylate-induced reduction in insulin secretion could also be related to the effects of salicylate on AMP protein kinase [42].

Beneficial effects were seen when an NF-κB inhibitor was administered immediately prior to the intraportal administration of islets [14], suggesting that acute inhibition has benefits. These studies indicate that while an acute inhibition may be beneficial in the few hours after implantation, a systemic chronic inhibition of NF-κB may be detrimental. In agreement with our study, McCall et al also showed that systemic administration of an NF-κB inhibitor over a period of weeks had no beneficial effects on islet transplantation outcome [43]. Inhibiting NF-κB systemically has been suggested to impair angiogenesis [44] and thus revascularization of the implanted islets may also be affected.

There is growing interest in the influence of NF-κB on β-cell function. Our in vitro studies on islets with activated NF-κB (βIKK) indicate that when evaluated for GSIS and various other parameters, they cannot be distinguished from normal islets. Unfortunately, because of breeding problems we were not able to study isolated islets from mice with inhibited NF-κB (βISR), but they had perfectly normal glucose tolerance. These results differ from those of Norlin et al, but their model was very different in that NF-κB activity was reduced by expression of a dominant active mutant IκBα under the Pdx1 promoter [45], which is turned on much earlier during β cell development. Hyperglycaemia seen in these mice may therefore reflect early embryonic effects of sustained NF-κB activity on islet development. Indeed, there was a 25% reduction in endocrine cell volume. The changes in islet function may have nothing to do with NF-κB inhibition because chronic hyperglycemia, even when very mild, is known to cause the type of β-cell dysfunction found in that study [46], [47]. Inhibition of insulin secretion was found with a very different approach of acute inhibition with an inhibitor of IκBα phosphorylation (Bay 11-7082) [48]. This again highlights the likely difference between acute and chronic inhibition of NF-κB as a potential explanation for these divergent results.

In summary, the current study draws attention to the complexities of regarding the activation state of NF-κB and how this activation state regulates the physiological function of β-cells. Our findings suggest that pancreatic β-cells can adapt to both chronic activation and inhibition of this important transcription factor with normal or near normal β-cell function.
